# Supplementary material for: Harnessing ChatGPT and GPT-4 for evaluating the rheumatology questions of the Spanish access exam to specialized medical training
Source: Sci Rep. 2023 Dec 13;13:22129. doi: 10.1038/s41598-023-49483-6 (PMC10719375; doi:10.1038/s41598-023-49483-6)
Supplement: Supplementary file 3 — Supplementary Information 3. [file 41598_2023_49483_MOESM3_ESM.docx]

**Harnessing ChatGPT and GPT-4 for Evaluating the Rheumatology Questions of the Spanish Access Exam to Specialized Medical Training**

Alfredo Madrid-García, Zulema Rosales-Rosado, Dalifer Freites-Nuñez, Inés Pérez-Sancristóbal, Esperanza Pato Cour, Chamaida Plasencia-Rodríguez, Luis Cabeza-Osorio, Lydia Abasolo-Alcázar, Leticia León-Mateos, Benjamín Fernández-Gutiérrez, Luis Rodríguez-Rodríguez

# Supplementary Figures


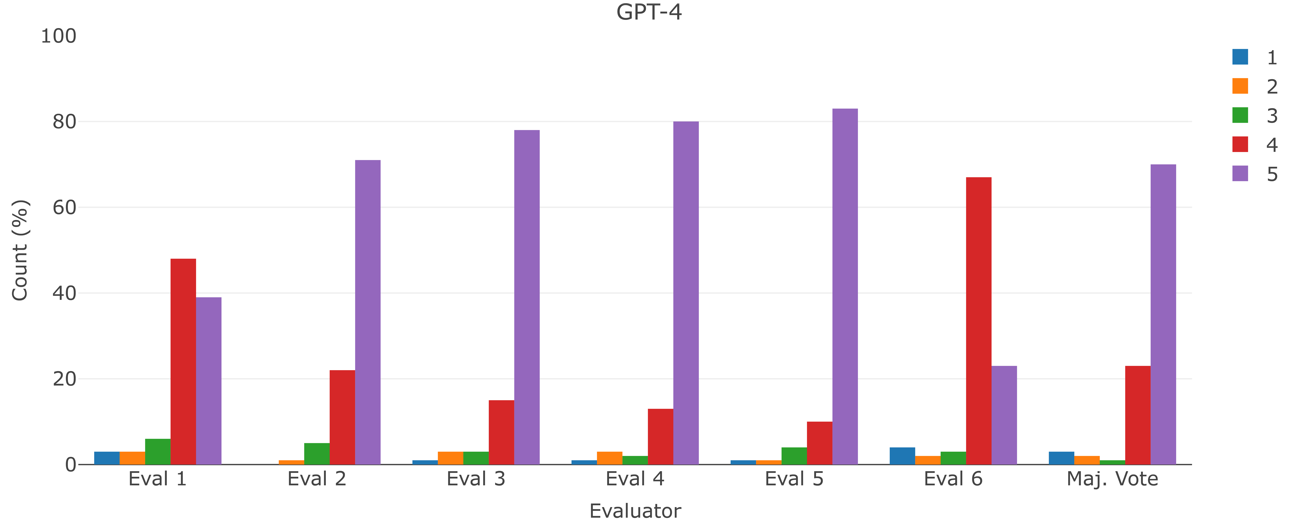


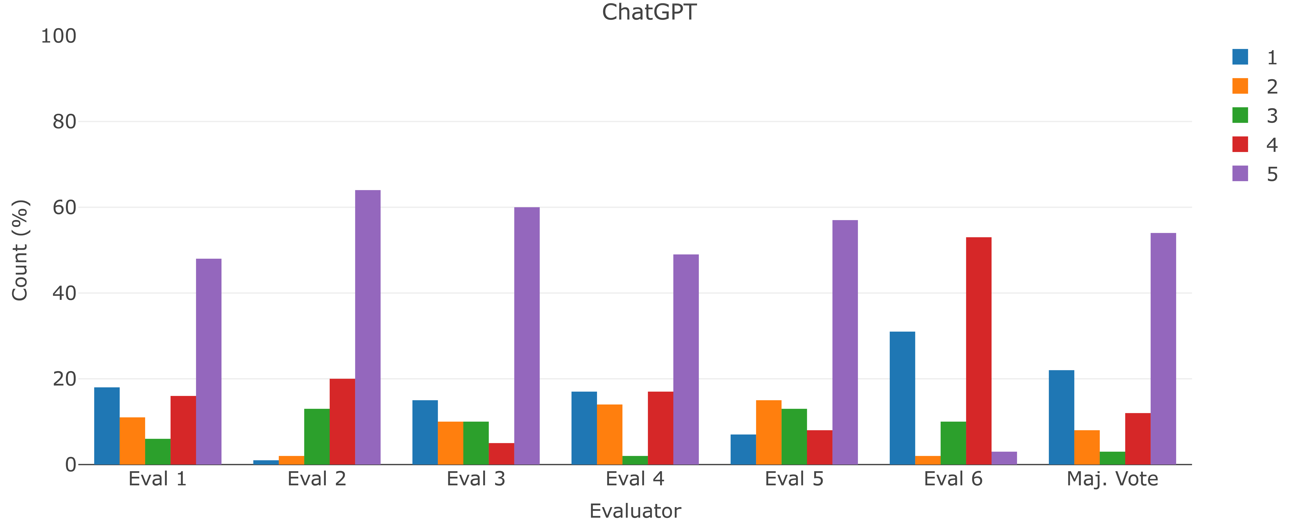


Supplementary Figure 1: Scores given by the evaluators to the clinical reasoning


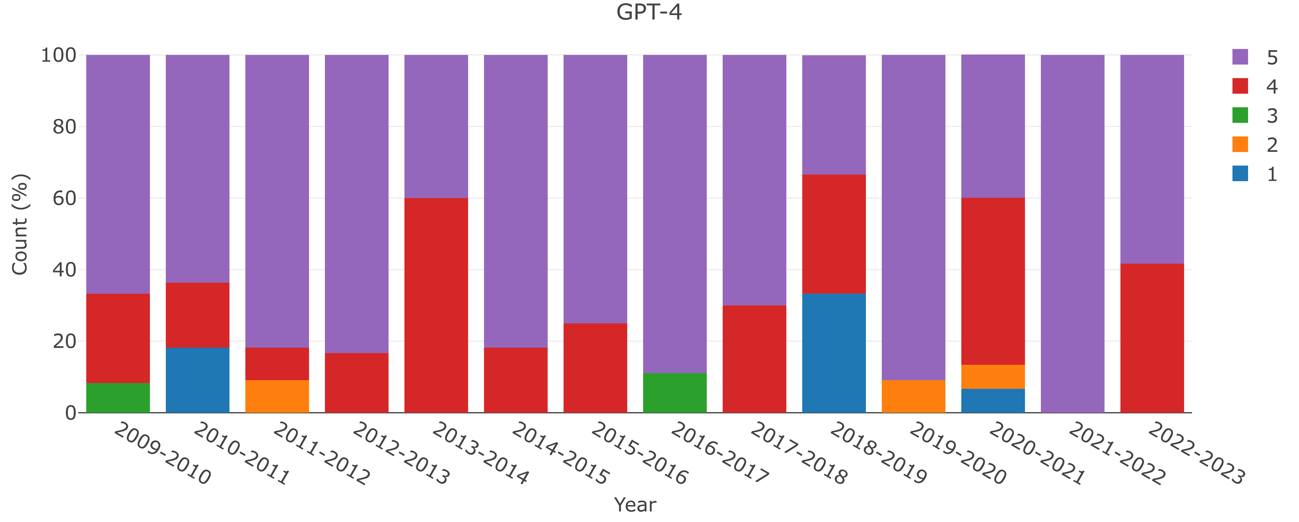


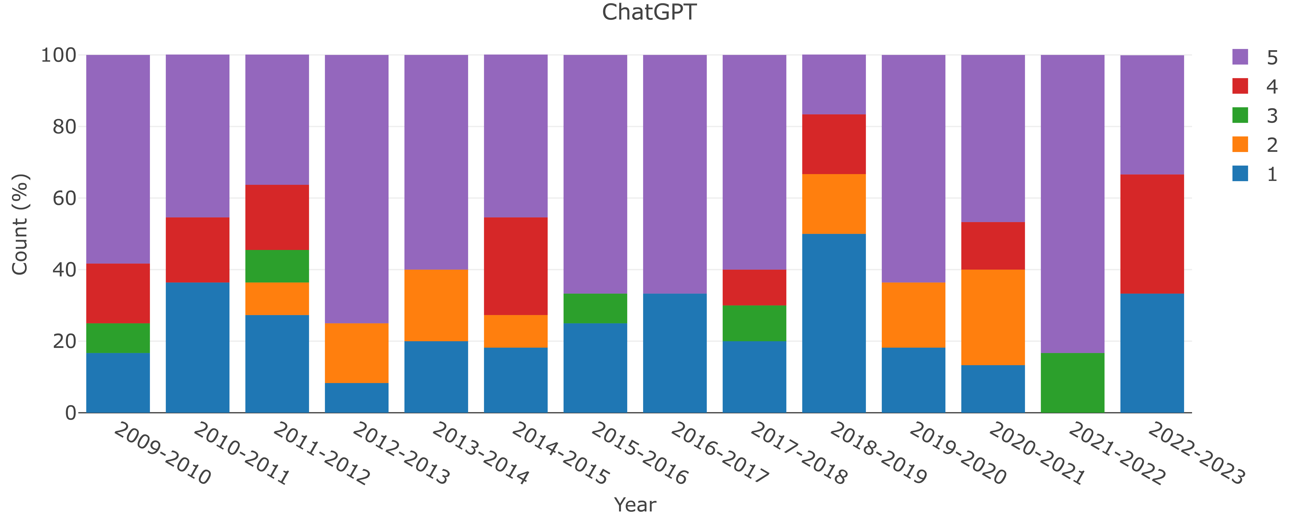


Supplementary Figure 2: Clinical reasoning score according to the year of the exam after taking the majority vote approach. In case of a tie, the worst score was chosen.


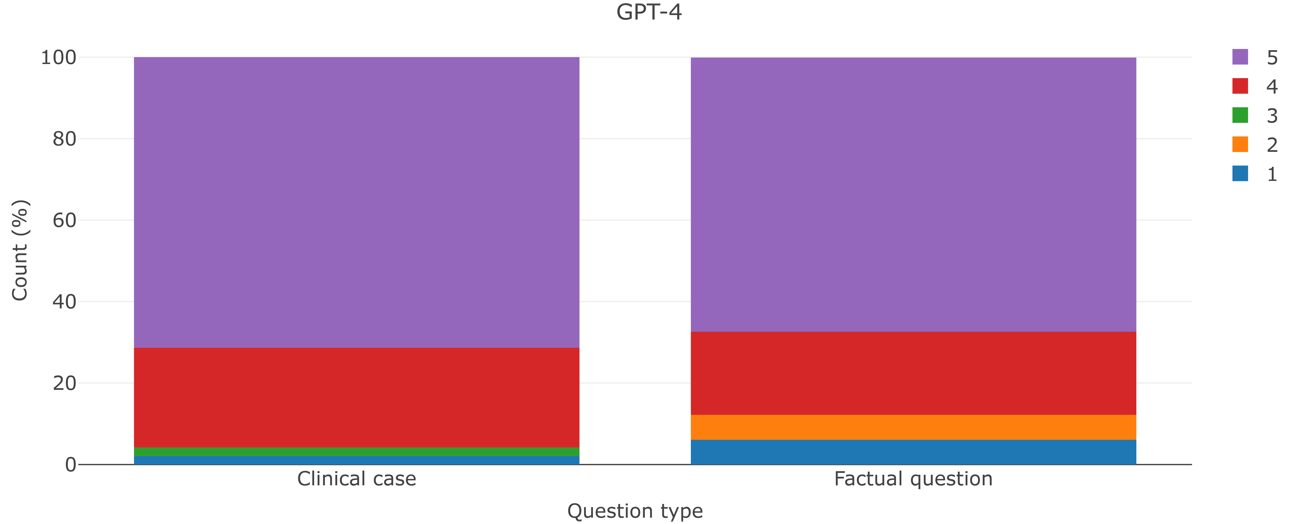


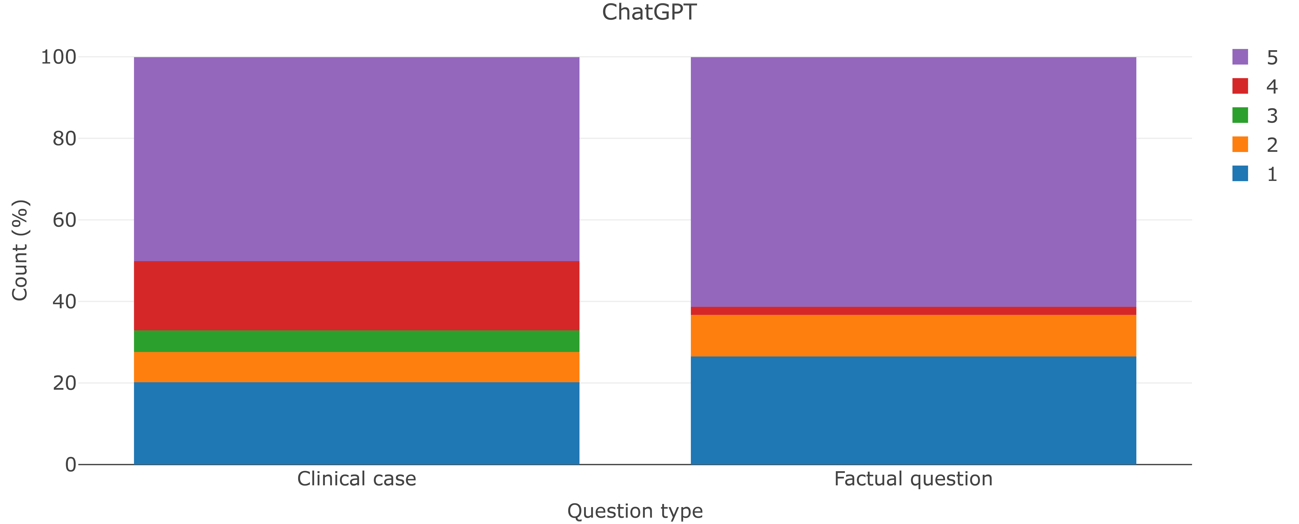


Supplementary Figure 3: Clinical reasoning score according to the type of the question after taking the majority vote approach. In case of a tie, the worst score was chosen


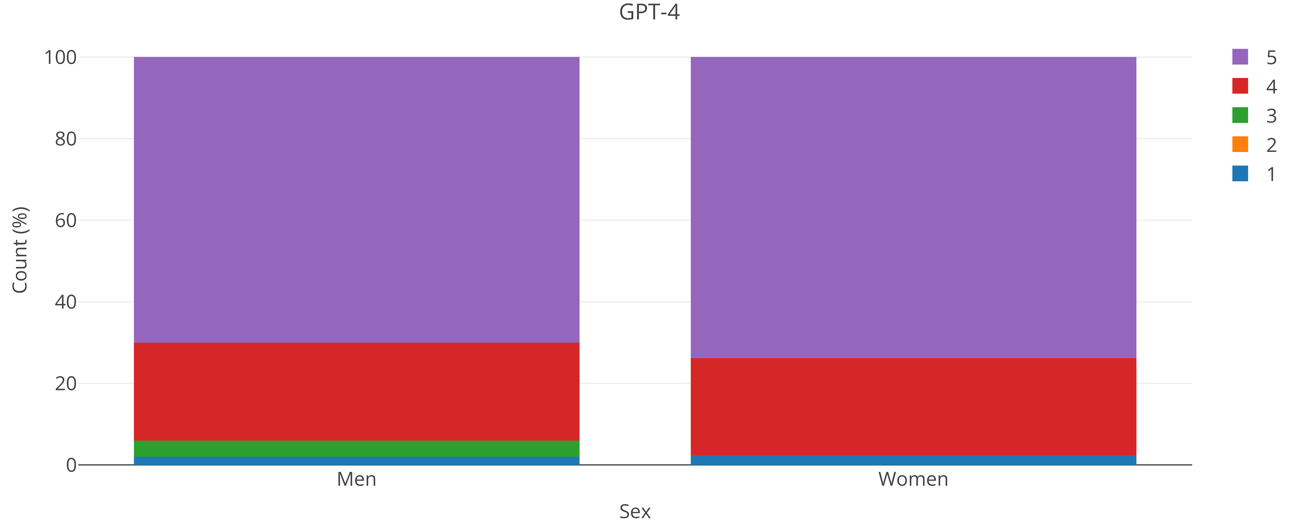


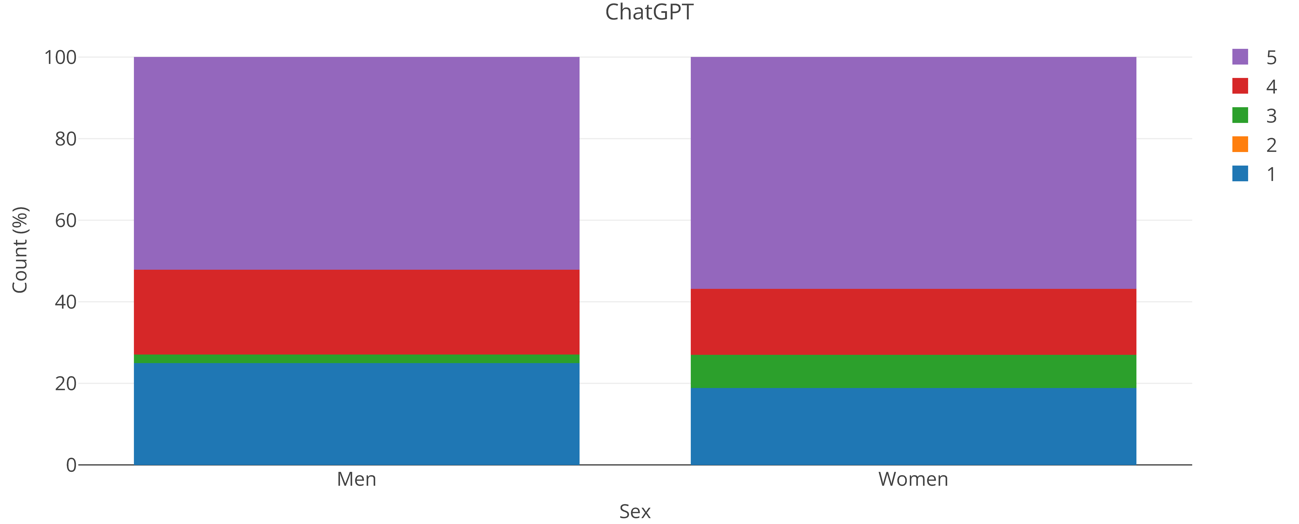


Supplementary Figure 4: Clinical reasoning score according to the sex after taking the majority vote approach. In case of a tie, the worst score was chosen

# Expert review process

# The evaluation process was conducted independently and unblinded. Each evaluator received the same documentation, and no attempt was made to hide the identity of the rest of evaluators. They worked independently without any consensus/meeting not agreed by the study organisers.

# There was a first round of assessment where evaluators 1 and 2 (i.e., MIR teacher and non-teacher) received the MIR exam questions and the ChatGPT/GPT-4 clinical reasoning in an excel document. During this phase, they were instructed to discuss the clinical reasoning for up to 30 questions in free-text format and assign scores ranging from 1 (strongly disagree) to 5 (strongly agree).

# Subsequently, the study designers met with the evaluators to discuss the evaluation process. At this meeting, it was agreed only to discuss in writing those questions that were necessary and to refine the evaluation system, agreeing on the creation of the scale shown in Table 1.

# The study was then expanded to include additional clinical reasoning assessments from MIR trainers and practicing rheumatologists. Evaluators 3 and 4, physicians from our hospital, along with evaluators 5 and 6, who were recruited through evaluator 1, joined the study.

# Rheumatologists 1, 5 and 6 were aware of each other's involvement in the study. Rheumatologists 1, 2, 3, 4 were aware of each other's involvement in the study.

# All participating rheumatologists, including evaluators 1 and 2, were provided with an Excel document containing the questions, ChatGPT/GPT-4's clinical reasoning, and the evaluation rubric. They were then requested to submit their ratings.

# After completing the question assessments, the evaluators filled out the free-text questionnaire.

# Evaluator agreement

Evaluators #1, #5 and #6 were, in addition to active rheumatologists, MIR training professors.

Supplementary Table 1: Number of questions with two or less points (negative) difference in the clinical reasoning of GPT-4 among evaluators.

| **Evaluator** | **1** | **2** | **3** | **4** | **5** | **6** |
| --- | --- | --- | --- | --- | --- | --- |
| **1** |  | 11 | 3 | 6 | 8 | 3 |
| **2** |  |  | 2 | 3 | 2 | 1 |
| **3** |  |  |  | 1 | 3 | 1 |
| **4** |  |  |  |  | 1 | 0 |
| **5** |  |  |  |  |  | 0 |

Supplementary Table 2: Number of questions with two or more points (positive) difference in the clinical reasoning of GPT-4 among evaluators.

| **Evaluator** | **1** | **2** | **3** | **4** | **5** | **6** |
| --- | --- | --- | --- | --- | --- | --- |
| **1** |  | 1 | 0 | 0 | 1 | 2 |
| **2** |  |  | 1 | 3 | 3 | 7 |
| **3** |  |  |  | 1 | 3 | 1 |
| **4** |  |  |  |  | 1 | 5 |
| **5** |  |  |  |  |  | 7 |

Supplementary Table 3: Number of questions with two or less points (negative) difference in the clinical reasoning of ChatGPT among evaluators.

| **Evaluator** | **1** | **2** | **3** | **4** | **5** | **6** |
| --- | --- | --- | --- | --- | --- | --- |
| **1** |  | 36 | 5 | 5 | 11 | 1 |
| **2** |  |  | 2 | 0 | 1 | 2 |
| **3** |  |  |  | 4 | 10 | 1 |
| **4** |  |  |  |  | 9 | 2 |
| **5** |  |  |  |  |  | 2 |

Supplementary Table 4: Number of questions with two or more points (positive) difference in the clinical reasoning of ChatGPT among evaluators.

| **Evaluator** | **1** | **2** | **3** | **4** | **5** | **6** |
| --- | --- | --- | --- | --- | --- | --- |
| **1** |  | 0 | 1 | 4 | 2 | 17 |
| **2** |  |  | 31 | 31 | 25 | 55 |
| **3** |  |  |  | 6 | 4 | 24 |
| **4** |  |  |  |  | 3 | 17 |
| **5** |  |  |  |  |  | 28 |

Supplementary Table 5: Agreement among all evaluators. Perfect agreement: no point of difference among evaluators. Strong agreement: one point of difference among evaluators. Medium agreement: two points of difference among evaluators. Low agreement: more than two points of difference among evaluators

|  |  | **ChatGPT** | | | |
| --- | --- | --- | --- | --- | --- |
|  | **Score** | **Low** | **Medium** | **Strong** | **Perfect** |
| **GPT-4** | **Low** | 5 | 0 | 0 | 0 |
|  | **Medium** | 3 | 5 | 8 | 0 |
|  | **Strong** | 19 | 33 | 61 | 2 |
|  | **Perfect** | 1 | 2 | 3 | 1 |

It is relevant to note that for all the questions where there was low agreement (5/5), GPT-4 failed to answer the questions. Whereas all but one in ChatGPT (28/29)

Supplementary Table 6: Median value of the average score for the clinical reasoning of the two evaluators with the largest differences

| **Setting** | **LLM model** | **Median value of the average score (Q1-Q3)** | **Evaluators** |
| --- | --- | --- | --- |
| Greater absolute difference between evaluators | GPT-4 | 4.5 (4.5-4.5) | #5, #6 |
| Smaller absolute difference between evaluators | GPT-4 | 5 (4.5-5) | #4, #5 |
| Greater absolute difference between evaluators | ChatGPT | 4 (2.75 - 4.5) | #2, #6 |
| Smaller absolute difference between evaluators | ChatGPT | 4.5 (2 - 4.5) | #1, #3 |

Supplementary Table 7: Clinical reasoning score of the errors according to the majority vote approach for ChatGPT and GPT-4

| **Clinical reasoning score** | **ChatGPT** | **GPT-4** |
| --- | --- | --- |
| 1 | 32 | 5 |
| 2 | 12 | 3 |
| 3 | 4 | 1 |

# Error analysis

The questions where GPT-4 failed are discussed below by #Reviewer 1:

**Question 26:** In scleroderma, in order to diagnose lung disease in its early stages, it is advisable to perform the following: Justify your answer

1. Functional respiratory tests and high-resolution CT scans every year.

2. Doppler echocardiogram and respiratory functional tests, only if the patient shows signs of respiratory and/or cardiac insufficiency.

3. Respiratory function tests and Doppler echocardiogram, both on an annual basis.

4. Respiratory function tests and 6-minute walk test.

**GPT-4 clinical reasoning:** Option 1, "High-resolution CT and respiratory function tests every year", is the most recommended option for early diagnosis of lung disease in patients with scleroderma.

Scleroderma is an autoimmune disease that can cause a variety of symptoms, including pulmonary fibrosis and pulmonary arterial hypertension, two of the most serious and common complications of the disease. Both can cause late symptoms and lead to a significant decrease in quality of life and survival. Functional respiratory tests (FFTs), which include spirometry, lung volumes and diffusion of carbon monoxide (DLCO), are useful for monitoring lung function in patients with scleroderma, as they can detect declining lung function before the patient develops symptoms.

On the other hand, high-resolution computed tomography (HRCT) is a very useful tool to assess lung anatomy and detect early changes in the lungs, such as fibrosis, which may not be evident on respiratory function tests. Therefore, regular (annual) performance of these tests may help to detect early lung involvement in patients with scleroderma and allow early intervention to treat and/or control disease progression. The other options are not as comprehensive and may not detect lung disease until significant damage has already occurred. For example, option 2 only suggests testing in the presence of symptoms, which could delay diagnosis. Option 3 does not include high-resolution CT scanning, which can detect changes in the lungs earlier than respiratory function tests. Option 4 does not include any imaging tests, which are essential for early diagnosis of lung disease.

**Discussion**: Although it is true that HRCT is more sensitive in detecting ILD, it is neither profitable nor safe to perform it annually in asymptomatic patients without diagnosed or suspected ILD; furthermore, in this option they do not check for pulmonary hypertension. However, option 3 performs respiratory function tests that are very sensitive to change along with Doppler ultrasound; both techniques are not invasive and are easy to perform, in addition to having good diagnostic yield for ILD and PHT; It is the least bad of the options provided

**Agreement:** Strong agreement among evaluators

**Question 30:** A 75-year-old man, hypertensive on treatment with enalapril (20 mg/day), consulted for deterioration of general condition, bitemporal headache and mandibular claudication of 3 weeks' duration. In addition, in the last few hours, he reported two episodes of amaurosis fugax of the left eye. Physical examination revealed thickening and absence of pulse in the left temporal artery. Analysis: CRP 6 mg/dl (VN <1); haemoglobin 10.5 g/dl; ESR 92 mm. Chest X-ray normal. Taking into account the most likely diagnosis, tick the correct option regarding treatment: Justify your answer.

1. Rituximab should be administered, as glucocorticoids have been relegated to second-line treatment due to their side effects.

2. Tocilizumab has been shown to be effective in reducing relapses and cumulative prednisone dose in more than 50% of patients.

3. In the presence of ischaemic symptoms, and in order not to worsen the patient's vascular risk, glucocorticoids at doses higher than 30 mg/day should be avoided.

4. In conjunction with glucocorticoid boluses of 1 g/day, infliximab should be used as a first-line drug for induction of remission.

**GPT-4 clinical reasoning:** The suggested clinical scenario is compatible with giant cell arteritis (GCA), also known as temporal arteritis. Classic symptoms of this disease include headache, mandibular claudication, and visual symptoms such as amaurosis fugax. Thickening and absence of temporal artery pulses on physical examination, along with laboratory test results showing inflammation (elevated CRP and elevated ESR), support this diagnosis. The correct option is "Along with glucocorticoid boluses of 1 g/day, infliximab should be used as a first-line drug for remission induction." (option 4). GCA is a medical emergency due to the risk of vision loss, and treatment with high-dose glucocorticoids (usually with prednisone) should be initiated immediately. The other options are not correct in the context of GCA: Although rituximab has been shown to be effective in some autoimmune diseases, it is not the first line of treatment for GCA, and glucocorticoids have not been relegated to second-line treatment. Tocilizumab, a monoclonal antibody against the interleukin-6 receptor, has shown promise in some studies for the treatment of GCA, but has not replaced the role of glucocorticoids as first-line therapy. Although glucocorticoids can have side effects, their use at high doses is critical in GCA to prevent vision loss. The dose will be adjusted and gradually reduced according to the patient's response to treatment. Please note that the dose of glucocorticoids may vary and should be determined by a physician based on the patient's specific clinical situation. Some experts may not start with 1 g/day boluses, but with high oral doses of prednisone.

**Discussion:** I believe the chat fails this question because it understands that in answer 4, correct answer, they are saying that the treatment is tocilizumab without a corticosteroid when all it says is that this treatment helps to reduce the accumulated dose of corticosteroid. The chat chooses option 1, the only answer that clearly states that GCA treatment is high-dose corticosteroids, ignoring the infliximab part.

**Agreement:** Strong agreement among evaluators

**Question 33:** With regard to temporal arteritis, mark the WRONG answer: Justify your answer.

1. It occurs mainly in white people over the age of 50 years.

2. Approximately a quarter of patients develop aneurysm or dilatation of the thoracic aorta.

3. Unilateral or bilateral vision loss is one of the most serious complications.

4. The peak incidence is between the sixth and seventh decade of life.

**GPT-4 clinical reasoning:** The incorrect answer is "Approximately one-fourth of patients develop thoracic aortic aneurysm or dilatation of the thoracic aorta." (option 2). Giant cell arteritis, also known as temporal arteritis, is an inflammatory disease that affects medium- and large-sized arteries, especially the temporal arteries, which are blood vessels in the temple area. Although it can sometimes affect the aorta and cause aortic aneurysms, this complication is much less common than the one indicated in option 2. According to studies, the prevalence of aortic aneurysm in patients with giant cell arteritis is variable, but is generally reported to be less than 20%.The other options are correct:

Giant cell arteritis is most common in Caucasians and those over 50 years of age. It is rare in people of other races and in younger people. Unilateral or bilateral vision loss can occur in giant cell arteritis due to optic nerve ischemia and is a serious complication that can lead to blindness if left untreated. The peak incidence of giant cell arteritis is usually between the sixth and seventh decade of life, although it can occur at any age in people over 50 years of age.

**Discussion**: This is a highly complex question; incorrect answer is 4 because incidence of GCA increases with age after 50 years; I believe the chat fails because this option is poorly written. Regarding the correct answer, number 2, symptomatic aneurysms appear in 15% of patients but asymptomatic aneurysms appear in a much higher percentage.

**Agreement:** Strong agreement among evaluators

**Question 41**: A 19-year-old woman came to the emergency department with symmetrical polyarthritis of small joints of the hands and feet, ankles and knees with fever of 39ºC for 24 hours. The most likely aetiology is: Justify your answer

1. Bacterial.

2. Autoimmune.

3. Microcrystalline.

4. Viral.

**GPT-4 clinical reasoning**: The most likely option is 2: "Autoimmune". The pattern of symmetrical arthritis, particularly in the small joints of the hands and feet, along with fever, suggests the possibility of an autoimmune disease, such as rheumatoid arthritis (RA). RA can occur in young people and often begins with symmetrical polyarthritis. Option 1, a bacterial infection, usually causes monoarticular or asymmetric arthritis, although it can affect multiple joints in cases of bacterial sepsis. Option 3, a microcrystalline arthritis (such as gout or pseudogout), is also usually monoarticular and is less common in such young people, unless there are predisposing factors such as certain metabolic disorders. Option 4, a viral infection, can cause arthritis, but is often accompanied by other symptoms such as rash or respiratory symptoms. Of course, further tests would be needed to confirm the diagnosis in this case, including laboratory tests and possibly imaging tests.

**Discussion:** The big mistake that the chat makes in this case is that it does not give importance to the duration of the condition (24 hours: acute polyarthritis). This concept is repeated repeatedly in the MIR examination: acute polyarthritis, except in some rare cases, is of viral origin.

**Agreement:** Low agreement among evaluators

**Question 42:** Which of the following radiological signs is NOT typical of ankylosing spondylitis: Justify your answer.

1. Squaring of the vertebral bodies.

2. Calcification of the anterior common vertebral ligament.

3. Sacroiliitis.

4. Dorsal kyphosis.

**GPT-4 clinical reasoning:** The correct answer is option 4: "Dorsal kyphosis". Ankylosing spondylitis is a chronic inflammatory disease that primarily affects the spine and sacroiliac joints. Typical radiological signs of the disease include sacroiliitis (inflammation of the sacroiliac joints), quadrature of the vertebral bodies and calcification of the anterior common spinal ligament, which can lead to the formation of a "bamboo spine". Dorsal kyphosis, however, is not a typical sign of ankylosing spondylitis. Although the disease can lead to postural changes, these are usually the result of compensatory hyperextension of the cervical and lumbar spine in response to limited mobility of the dorsal spine, rather than kyphosis per se. Therefore, the presence of dorsal kyphosis is not a typical finding in ankylosing spondylitis.

**Discussion**: I think here the chat is making a misconception since what calcifies is the annulus fibrosus and not the anterior ligament.

**Agreement:** Low agreement among evaluators

**Question 93:** Which of the following methods do you consider to be the most effective in preventing hip fracture? Please justify your answer.

1. Oestrogens.

2. Calcium and Vitamin D.

3. Bisphosphonates.

4. Physical activity.

5. Calcitonin.

**GPT-4 clinical reasoning:** Prevention of hip fracture involves several factors, but among the options provided, bisphosphonates have been shown to be an effective treatment to prevent hip fracture in patients with osteoporosis. Bisphosphonates work by inhibiting bone resorption, that is, the process by which bone tissue is broken down and released into the bloodstream. Consequently, this reduces the loss of bone density and may reduce the risk of fractures. Physical activity is also an important component in preventing hip fracture, as regular exercise, especially weight-bearing exercise, can help maintain bone density and strength. Calcium and vitamin D are critical to bone health and may help prevent bone loss, but their efficacy in preventing hip fracture has not been shown to be as strong as bisphosphonates. Estrogens have been used in the past for the prevention and treatment of osteoporosis, but their use has declined due to associated risks, such as breast cancer and cardiovascular events. Calcitonin is a drug used to treat osteoporosis, but it is not first-line due to its lower efficacy compared to other treatments. Importantly, the choice of treatment should be based on an individualized assessment of the patient, taking into account factors such as age, general health, fracture risk and the presence of osteoporosis. In this question there is an effective non-pharmacological measure (physical activity) and two pharmacological measures with also demonstrated effectiveness (bisphosphonates and estrogens). Since there are two effective pharmacological measures, it is logical to think that the correct response is the non-pharmacological one. Maybe the chat fails this question because it does not have an exam technique.

**Discussion**: In this question there is an effective non-pharmacological measure (physical activity) and two pharmacological measures with also demonstrated effectiveness (bisphosphonates and estrogens). Since there are two effective pharmacological measures, it is logical to think that the correct response is the non-pharmacological one. Maybe the chat fails this question because it does not have an exam technique.

**Agreement:** Strong agreement among evaluators

**Question 105:** Of the following complementary tests, which one would you indicate in the case of a patient with rheumatoid arthritis presenting with nephrotic syndrome: Justify your answer.

1. Rectal biopsy.

2. Renovesical ultrasound.

3. Intravenous pyelography.

4. Isotopic renogram.

5. Renal echo-Doppler

**GPT-4 clinical reasoning:** The most appropriate option in this case would be a renal biopsy, although it is not mentioned in the options given. Renal biopsy allows identification of the type of renal lesion, which is critical in determining the appropriate treatment. However, among the options provided, none seems to be the most appropriate for a patient with rheumatoid arthritis presenting with nephrotic syndrome. Rectal biopsy: This is not useful in this case, as it does not provide information on the status of the kidneys. Renal renovesical ultrasound: This test may reveal renal abnormalities of size or structure, but would not provide detailed information about the cause of a nephrotic syndrome. Intravenous pyelography: This test is used to evaluate the structure of the urinary system, but does not provide information about kidney function at the glomerular level, which is where nephrotic syndrome usually occurs. Isotopic renogram: This test evaluates kidney function, but would not provide detailed information about the cause of a nephrotic syndrome. Renal echo-Doppler: This test evaluates blood flow in the kidneys and could detect renal perfusion problems, but would not be the most appropriate test to evaluate nephrotic syndrome. In summary, of the options provided, none provides an adequate assessment of nephrotic syndrome. In clinical practice, renal biopsy would be the most appropriate option for a patient with rheumatoid arthritis presenting with nephrotic syndrome.

**Discussion**: The chat fails this question because it approaches it completely wrongly, in addition to a failure in examination technique. It focuses on studying the patient's kidney problem but does not put it in the clinical context, that is, a patient with a chronic inflammatory disease (rheumatoid arthritis) who presents a nephrotic syndrome. In these cases, the priority is to rule out secondary amyloidosis, again a frequently asked concept in the MIR examination.

**Agreement:** Low agreement among evaluators

**Question 107:** Mark the FALSE answer about the characteristics of a synovial joint: Justify your answer.

1. Hyaline articular cartilage consists mainly of collagen.

2. The extracellular matrix proteoglycans of hyaline cartilage are macromolecules consisting of proteins and glycosaminoglycans.

3. The chondrocyte lives in an acidotic and hypoxic environment and uses anaerobic glycolysis for energy production.

4. Type A synoviocytes are macrophage-like, phagocytic.

5. An enthesis is the region where a tendon, ligament or joint capsule attaches directly or indirectly to bone.

**GPT-4 clinical reasoning:** The FALSE statement is the third one: 3. The chondrocyte lives in an acidotic and hypoxic environment and uses anaerobic glycolysis to produce energy. Justification: Chondrocytes, which are the cells of cartilage, live in an avascular environment, so they can adapt to low-oxygen conditions. However, to say that they live in an "acidotic environment" is not correct. Furthermore, although they can resort to anaerobic glycolysis due to lack of oxygen supply, their main method of energy production is aerobic glycolysis when there is sufficient oxygen supply. Although chondrocytes can survive under hypoxic conditions, this is not their preferred state nor is it the normal state of the articular cartilage environment. In general, the cell has a limited capacity for anaerobic glycolysis and would prefer aerobic glycolysis when possible. Therefore, this statement is incorrect or at least misleading.

**Discussion:** I don’t really understand why the chat fails this question, I guess it got his information from the wrong place.

**Agreement:** Low agreement among evaluators

**Question 109:** A 70-year-old man is diagnosed with giant cell temporal arteritis. Treatment should be started with: Justify your answer.

1. Prednisone 20 mg daily.

2. Prednisone 40-60 mg daily.

3. Prednisone 40-60 mg each day and cyclophosphamide.

4. Prednisone 40-60 mg each day, calcium and vitamin D and bisphosphonates.

5. Oral cyclophosphamide.

**GPT-4 clinical reasoning:** Giant cell arteritis, also known as temporal arteritis, is an inflammatory disease that mainly affects the arteries of the head, particularly the temporal arteries. This disease is most common in people over the age of 50 and can cause a number of symptoms, such as headache, fever, weight loss and, in severe cases, loss of vision. Option 2, prednisone 40-60 mg every day, is generally the recommended initial treatment for giant cell arteritis. Corticosteroids, such as prednisone, are potent anti-inflammatory drugs that can help reduce inflammation in the arteries and relieve the symptoms of this disease. In addition, option 4, prednisone 40-60 mg each day, calcium and vitamin D, and bisphosphonates, may also be a reasonable treatment option. Long-term corticosteroids can have side effects, including bone loss. Therefore, it is advisable to take calcium and vitamin D supplements and possibly bisphosphonates to help protect bone health during long-term corticosteroid therapy. Cyclophosphamide, an immunosuppressive agent, is generally not used as a first-line treatment for giant cell arteritis unless the patient does not respond to corticosteroid therapy or if the disease is particularly severe. Therefore, in most cases, option 2 or 4 would be recommended. However, it is always important to consider the individual circumstances of each patient, including any other health conditions they may have and the possible side effects of the drug.

**Discussion**: I believe the chat fails this question again due to exam technique; when faced with two identical options, you must choose the most complete one (in this case the option that, in addition to administering corticosteroids, also administers osteoporosis prophylaxis).

**Agreement:** Low agreement among evaluators

# LLM comparison

The accuracy of BARD by Google, version 11^th^ July 2023, and Claude 2 by Antrophic, version 11^th^ July 2023, was compared against GPT-4 and ChatGPT. Only the accuracy of these two models were measured and not their clinical reasoning.

As it was done with the OpenAI solutions, the prompts were introduced in Spanish, exactly as we did for ChatGPT and GPT-4.


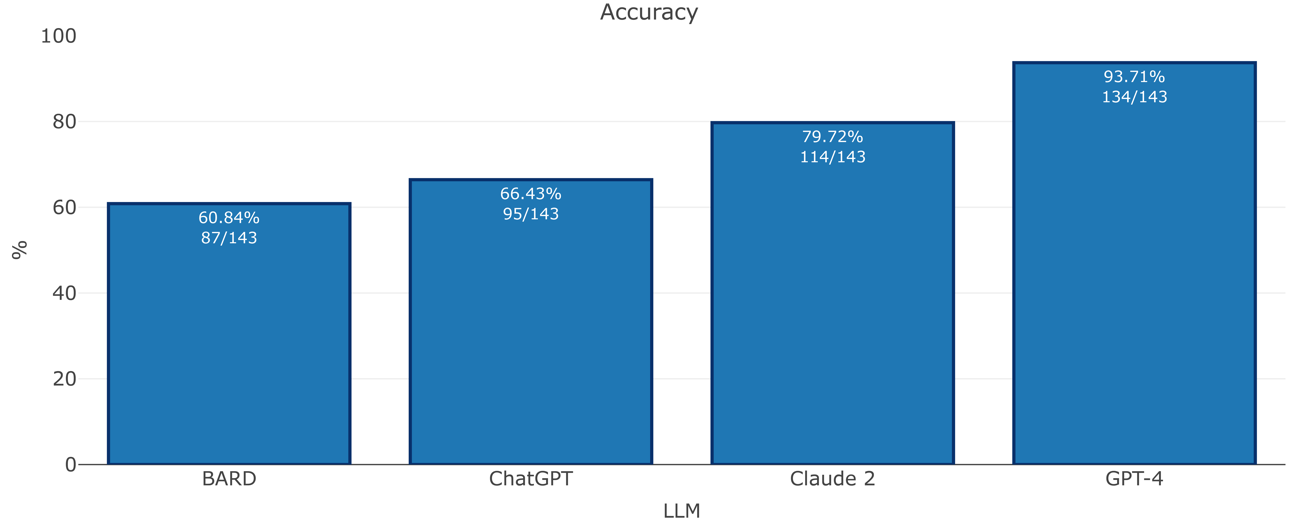


Supplementary Figure 5: Accuracy of BARD, ChatGPT, Claude 2 and GPT-4 in a dataset of 143 rheumatology questions
