# Supplementary material for: Harnessing ChatGPT and GPT-4 for evaluating the rheumatology questions of the Spanish access exam to specialized medical training
Source: Sci Rep. 2023 Dec 13;13:22129. doi: 10.1038/s41598-023-49483-6 (PMC10719375; doi:10.1038/s41598-023-49483-6)
Supplement: Supplementary file 4 — Supplementary Information 4. [file 41598_2023_49483_MOESM4_ESM.docx]

## Supplementary Material: "Questionnaire for evaluating the use of language models in rheumatology"

# English questionnaire

**1. Based on the clinical reasoning you have evaluated; do you think that the use of ChatGPT/GPT-4 could be useful as a content generator for the medical student who is preparing rheumatology-related content for the MIR exam?**

**Evaluator 1:** Yes, it could, but with supervision of the content by a subject specialist as most of the answers need "polishing".

**Evaluator 2:** I think ChatGPT-4 is useful as a content generator for the medical student. I think it is useful after the basic training rounds, I consider it valuable in the final stage of the student's training. Its supervised use with the subject trainers is recommended. Regarding ChatGPT as a content generator, it requires more guidance/supervision in its use.

**Evaluator 3:** I think so, as long as, it was supervised by a specialist in the subject or a trainer/teacher of this type of exams, but of course, as a method for practicing, I think it would be useful.

**Evaluator 4:** I think it could be useful as a tool to help generate content, but always with supervision of the content by a specialist in the field. For me it would be useful to use GPT-4, I would not use ChatGPT, in some cases to consult something and with a lot of supervision.

**Evaluator 5:** Yes, although it is true that it lacks training and supervision, especially GPT-4 can be useful.

**Evaluator 6:** Based on the hit rate it would be possible to use GPT-4 in this sense, not so much ChatGPT.

**2. Do you consider that the language used by both systems is appropriate for a medical student, does it contain/lack technicalities?**

**Evaluator 1:** It is not bad, for a MIR candidate it may be adequate, although I find it excessively colloquial, and it lacks technicalities. However, the answers are very well understood.

**Evaluator 2:** The language used in the ChatGPT/GPT-4 seems to me to be correct and appropriate for a medical student. The technicality of the content is just right to get the message across.

**Evaluator 3:** Sometimes it can be too colloquial, but in general, I think it is adequate for a MIR candidate, the explanations are usually very well understood.

**Evaluator 4:** It seems to me to be appropriate language for a recently finished doctor who is going to take an MIR exam. It is possible that the ChatGPT uses somewhat colloquial language.

**Evaluator 5:** Although some answers may be lacking in technicalities, the answers were well understood and for a MIR candidate the level could be adequate.

**Evaluator 6:** No, the language is not very academic for medical students.

**3. Are the justifications/reasoning of the ChatGPT/GPT-4 models clear, straightforward, and understandable?**

**Evaluator 1:** Chat-GPT's justifications are perhaps longer and more complete, but it fails more often so it is not applicable; GPT-4 gives more scientifically sound answers and is perhaps less broad, more direct.

**Evaluator 2:** In general, the justifications and reasoning in both models are clear and understandable. I consider ChatGPT-4 to be more direct in content.

**Evaluator 3:** Apart from a few cases, which could have been a little too concise and particularly convoluted, in general, I found the explanations to be well developed and well structured.

In particular, I felt that GPT-4 developed them better from a scientific point of view.

**Evaluator 4:** I find them understandable in 95% of cases or more, they are well structured and the documentation they use seems to be correct. Sometimes the justifications are a bit lengthy.

**Evaluator 5:** In general, although both systems give long justifications, my impression is that the GPT-4 ones are more scientifically sound and somewhat more straightforward and understandable than the ChatGPT ones.

**Evaluator 6:** The justifications are quite well-reasoned, even at great length in many cases.

**4. Do ChatGPT/GPT-4 models show awareness of the limitations and scope of their knowledge, avoiding speculation or incorrect answers when there is insufficient information?**

**Evaluator 1:** Not much, they always try to justify their answer (often incorrect) with whatever, without any criteria. In their favor, it must be said that at the end they always put the phrase that it is necessary to consult a doctor and that it is necessary to assess each individual case.

**Evaluator 2:** Not entirely, sometimes the content of the justifications/answers lacks reflection or debate. We found some questions with correct answers, not entirely well developed in their justification. The opposite is also observed (ChatGPT), where an incorrect answer is generated and the reasoning contains erroneous information as if it were correct. Students should approach their answers with caution.

**Evaluator 3:** Not always, sometimes, especially in the case of ChatGPT, incorrect answers are given and yet attempts are made to justify and explain even when the criteria on which they are based are not properly documented or up to date.

**Evaluator 4:** No, especially ChatGPT. It gives wrong answers and justifies them with wrong information as if they were correct.

**Evaluator 5:** Above all, GPT-4 is the system that manages to get it most right and to make the most accurate reasoning in my opinion.

**Evaluator 6:** Mostly they give an answer based on their knowledge, which may be wrong in some cases.

**5. As a rheumatology specialist, what application do you think ChatGPT/GPT4 could have, or what use would you make of it as a practitioner?**

**Evaluator 1:** They seem to me to be tools that can make it easier and quicker to create teaching material, but they need the supervision of a professional to check both the content and the language.

**Evaluator 2:** I consider it useful as an additional tool for the medical specialist. It could be applied in medical diagnosis, for reading medical records, analysing symptoms, analysis and imaging tests and for educational purposes. With the eventual development of the tool, GPT-4 has the potential to transform medical care and improve patient care. However, its implementation should be treated with caution, taking into account ethical, legal and practical issues.

**Evaluator 3:** Mainly, I think they could be used on the one hand, for teaching (creation of materials, guides, help with exams) and, on the other hand, it can be a tool that can help in specific clinical doubts as long as we are aware that it would only be a first approximation, that is, accessing the scientific information available in a faster and more concise way (similar to an "Uptodate") but always being the content supervised and taking into account by the specialists, its scope and limitations.

**Evaluator 4:** I think it could be useful as a tool to help in clinical doubts for a first approach, to supervise the response and to access scientific information in a more targeted way. It can also help to create scientific and teaching material but always with supervision.

**Evaluator 5:** As a professional, I believe that these systems can help us to speed up the creation of teaching material (for classes, exams, gamification...) but with the supervision of a professional who reviews the content, which is crucial at this time.

**Evaluator 6:** GPT4 is much more accurate and could be useful for generating questions and evaluations for courses/lectures/sessions.

**6. In general terms, what weaknesses/limitations have you identified in the answers provided by ChatGPT/GPT-4 (maximum of 3)?**

**Evaluator 1:**

- Their reasoning is different from that of teachers and clinicians, which causes them to fail the most difficult questions because they often need exam technique to answer them or more in-depth reasoning.
- It seems that the sources from which they take the information are not very scientific, no reference is made in any case to EULAR/ACR...
- They justify the unjustifiable with a reasoning that you could come to believe, even if it is wrong, that is worrying.

**Evaluator 2:**

- It seems to me a limitation not to acknowledge the source or bibliographic reference of the justification or reasoning. The model may generate answers based on generic knowledge rather than specific medical protocols or classification criteria, for example.
- From a training point of view, medical students preparing for the MIR exam, in its use there should be a process in which experts can review the content.

**Evaluator 3:**

- Sometimes explanations too convoluted and not very concise and brief.
- Explanations sometimes too informal, particularly ChatGPT
- Outdated information and not based on adequate sources (ACR, EULAR, rheumatology books, etc...).

**Evaluator 4:**

- Lacks awareness of errors and justifies them as correct (especially ChatGPT).
- Language is sometimes unscientific, more so in ChatGPT, and does not seem to base answers on established classification criteria.

**Evaluator 5:** In general, I have found that exceptions or difficult cases are more likely to escape ChatGPT than GPT-4. That is, cases that fall outside the norm are more difficult to discern the appropriate response.

**Evaluator 6:**

- Unscientific language in some cases
- Excessively long and exhaustive explanations

**7. In general terms, what strengths have you identified in the answers provided by ChatGPT/GPT-4 (maximum of 3)?**

**Evaluator 1:** They provide a lot of information quickly and with training could probably be very useful at this time for the general public rather than the specialist.

**Evaluator 2:**

- The answers, generated quickly, produce a well-structured text.
- The language used is correct and understandable.

**Evaluator 3:**

- Speed and convenience
- Ease of access to information, especially better with GPT-4 which has more hits and better reasoning of options.
- Understandable language.

**Evaluator 4:** More strengths with the GPT-4 which seems "more scientific" and gets most of the questions right, few errors. The language it uses is understandable.

**Evaluator 5:**

- Chat-GPT: reasons between true and false in general, understandable language
- Chat-GPT4: more hits, understandable, reasoning between true and false in general

**Evaluator 6:**

- Very high percentage of correct answers to the questions.
- Up-to-date bibliography

# Spanish questionnaire

1. **En base al razonamiento clínico que has evaluado, ¿crees que el uso de ChatGPT/GPT-4 podría ser de utilidad como generador de contenido para el estudiante de medicina que está preparando los contenidos relacionados con reumatología en el examen MIR?**

**Evaluador 1:** Sí podría, pero con supervisión del contenido por parte de un especialista en impartir la asignatura puesto que la mayoría de las respuestas necesitan un “pulido”.

**Evaluador 2:** Considero que el ChatGPT-4, si es útil como generador de contenido para el estudiante de medicina. Creo que es útil tras las rondas formativas de base, lo considero valioso en la etapa final del entrenamiento del estudiante. Su uso supervisado con los preparadores de la asignatura es recomendable. Respecto al ChatGPT como generador de contenido, requiere de una mayor guía/supervisión en su uso.

**Evaluador 3:** Creo que sí, siempre que estuviera supervisado por algún especialista de la materia o preparador/profesor de este tipo de exámenes, pero desde luego, como método para practicar si le veo utilidad.

**Evaluador 4:** Creo que si podría ser útil como una herramienta de ayuda para generar contenido, pero siempre con supervisión del contenido por parte de un especialista en la materia. Para mí sería útil el uso de GPT-4, el ChatGPT no lo utilizaría, en algún caso para consultar algo y con mucha supervisión.

**Evaluador 5:** Sí, aunque es cierto que le falta entrenamiento y supervisión, sobre todo GPT-4 puede ser de utilidad.

**Evaluador 6:** Basándonos en el porcentaje de aciertos sería posible utilizar GPT-4 en este sentido, no tanto ChatGPT

1. **¿Consideras que el lenguaje empleado por ambos sistemas es el adecuado para un estudiante de medicina, contiene/carece tecnicismos?**

**Evaluador 1:** No está mal, para un opositor MIR puede ser adecuado, aunque me resulta excesivamente coloquial y le faltan tecnicismos. Pero son respuestas que se entienden muy bien.

**Evaluador 2:** El lenguaje empleado en el ChatGPT/GPT-4 me parece correcto y adecuado para un estudiante de medicina. El tecnicismo del contenido es el justo para trasmitir el mensaje.

**Evaluador 3:** A veces pueda resultar demasiado coloquial, pero en general, sí me parece adecuado para un opositor MIR, las explicaciones suelen entenderse muy bien.

**Evaluador 4:** Me parece que un lenguaje apropiado para un médico recién acabado que va a hacer un examen MIR. Es posible que el ChatGPT utilice un lenguaje un poco coloquial.

**Evaluador 5:** Aunque algunas respuestas pueden carecer de tecnicismos, las respuestas se entendían bien y de cara a un opositor MIR el nivel podría ser el adecuado.

**Evaluador 6:** No, el lenguaje es poco académico para los estudiantes de Medicina

1. **¿Las justificaciones/razonamiento de los modelos ChatGPT/GPT-4 son claras, directas y comprensibles?**

**Evaluador 1:** Quizá las justificaciones del ChatGPT son más largas y completas, pero falla con mayor frecuencia por lo que muchas veces no se pueden aplicar; el GPT-4 da respuestas más acertadas científicamente y quizá son menos amplias, más directas.

**Evaluador 2:** En general las justificaciones y razonamientos en ambos modelos son claras y comprensibles. Considero que el ChatGPT-4 es más directo en el contenido.

**Evaluador 3:** Salvo en algunos casos, que podían resultar algo poco concisas y especialmente enrevesadas, en general, me parece que están bien desarrolladas y estructuradas las explicaciones.

Concretamente, me dio la sensación de que GPT-4 las desarrolla mejor desde un punto de vista científico.

**Evaluador 4:** Si me parecen comprensibles en el 95% de los casos o más, están bien estructuradas y parece que la documentación que utilizan es correcta. A veces las justificaciones son un poco extensas.

**Evaluador 5:** En general, aunque los dos sistemas de ChatGPT dan justificaciones largas, mi impresión es que las de GPT-4 son más acertadas científicamente y algo más directas y comprensibles que las de ChatGPT.

**Evaluador 6:** Las justificaciones son bastante razonadas, incluso de gran extensión en muchos de los casos.

1. **¿Los modelos ChatGPT/GPT-4 muestran conciencia de las limitaciones y alcance de su conocimiento, evitando especulaciones o respuestas incorrectas cuando no tiene suficiente información?**

**Evaluador 1:** No mucho, siempre intentan justificar su respuesta (muchas veces incorrecta) con lo que sea sin un criterio. En su favor hay que decir que al final siempre ponen la coletilla de que hay que consultar con un médico y que es necesario valorar cada caso concreto.

**Evaluador 2:** No del todo, en ocasiones el contenido de las justificaciones/respuestas carecen de reflexión o debate. Encontramos algunas preguntas con respuestas correctas, no del todo bien desarrolladas en su justificación. También se observa lo contrario (ChatGPT), en la que se genera una respuesta incorrecta y el razonamiento contiene información errónea como si fuera correcta. Los estudiantes deben abordar sus respuestas con precaución.

**Evaluador 3:** No siempre, en algunas ocasiones, sobre todo en el caso de ChatGPT, se dan respuestas incorrectas y aun así, se intenta justificar y explicar aún cuando los criterios en los que se basan no están debidamente documentados o actualizados.

**Evaluador 4:** No, sobre todo el ChatGPT. Da respuestas incorrectas y las justifica con información errónea como si fuera correcta

**Evaluador 5:** Sobre todo es GPT-4 el sistema que consigue acertar más y hacer un razonamiento más acertado en mi criterio.

**Evaluador 6:** Mayoritariamente dan una respuesta basada en sus conocimientos, que pueden ser erróneos en algunos casos.

1. **Como especialista en reumatología, que aplicación crees que podría tener ChatGPT/GPT4, o que uso le darías tu como profesional.**

**Evaluador 1:** Me parecen herramientas que pueden hace más fácil y rápido crear material docente, pero necesitan la supervisión de un profesional que revise tanto el contenido como el lenguaje.

**Evaluador 2:** Considero que es útil como una herramienta más para el médico especialista. Podría ser aplicado en el diagnóstico médico, para leer registros médicos, analizar síntomas, análisis y pruebas de imagen y con fines educativos. Con el eventual desarrollo de la herramienta, el GPT-4 tiene el potencial de transformar la atención médica y mejorar la atención al paciente. No obstante, su implementación debe tratarse con cautela y teniendo en cuenta las cuestiones éticas, legales y prácticas

**Evaluador 3:** Principalmente, creo que se podrían utilizar por un lado, para docencia (creación de materiales, guías, ayudar con exámenes) y, por otro lado, puede ser una herramienta que puede ayudar en dudas clínicas concretas siempre y cuando seamos conscientes de que sería solo una primera aproximación, es decir, acceder a la información científica disponible de una forma más rápida y concisa *(similar a un “Uptodate”)* pero siempre siendo el contenido supervisado y teniendo en cuenta por parte de los especialistas, su alcance y limitaciones.

**Evaluador 4:** Creo que podrá ser útil como herramienta de ayuda en dudas clínicas para una primera aproximación, supervisar la respuesta y acceder a la información científica de forma más dirigida. También puede ayudar a crear material científico y docente pero siempre con supervisión

**Evaluador 5:** Como profesional considero que estos sistemas pueden ayudarnos a agilizar la creación material docente (para clases, exámentes, gamificación…) pero con la supervisión de un profesional que revise el contenido, lo cual en estos momentos es crucial.

**Evaluador 6:** GPT4 es mucho más precisa y podría tener utilidad para la generación de preguntas y evaluaciones de cara a cursos/ponencias/sesiones

1. **¿En rasgos generales, qué debilidades/limitaciones has identificado en las contestaciones proporcionadas por ChatGPT/GPT-4 (máximo de 3)?**

**Evaluador 1:**

- Su razonamiento es diferente del que hacemos los docentes y los clínicos lo que le hace fallar las preguntas más difíciles porque muchas veces necesitan de técnica de examen para ser contestadas o de un razonamiento más profundo.
- Parece que las fuentes de donde toman la información son poco científicas, no se hace referencia en ningún caso a EULAR/ACR….
- Justifican lo injustificable con un razonamiento que podrías llegar a creerte, aunque sea erróneo, eso es preocupante.

**Evaluador 2:**

- Me parece una limitación no reconocer la fuente o referencia bibliográfica de la justificación o razonamientos. El modelo puede generar respuestas basadas en conocimientos genéricos en lugar del protocolos médicos específicos o criterios de clasificación, por ejemplo.
- Desde el punto de vista formativo, estudiantes de medicina que está preparando el examen MIR, en su uso debe haber un proceso en el que los expertos puedan revisar el contenido.

**Evaluador 3:**

- A veces, explicaciones demasiado enrevesadas y poco concisas y breves.
- Explicaciones demasiado informales, en ocasiones, sobre todo ChatGPT.
- Información desactualizada y no basada en fuentes adecuadas (ACR, EULAR, libros de Reumatología, etc.)

**Evaluador 4:**

- No tiene conciencia de los errores y los justifica como si fueran correctos (sobre todo ChatGPT).
- El lenguaje en ocasiones es poco científico, más en ChatGPT.
- No parece basar las respuestas en criterios de clasificación establecidos.

**Evaluador 5:** En general, he detectado que las excepciones o casos difíciles se pueden escapar sobre todo a ChatGPT más que al GPT-4. Es decir, los casos que se salen de la norma son más difíciles de discernir la respuesta adecuada.

**Evaluador 6:**

- Lenguaje poco científico en algún caso
- Explicaciones demasiado largas y exahustivas

1. **¿En rasgos generales, qué fortalezas has identificado en las contestaciones proporcionadas por ChatGPT/GPT-4 (máximo de 3)?**

**Evaluador 1:** Proporcionan mucha información de manera rápida y probablemente con un entrenamiento podrían llegar a ser muy útiles en estos momentos más para el público general que para el especializado

**Evaluador 2:**

- Las contestaciones, son generadas con rapidez, generan un texto bien estructurado.
- El lenguaje utilizado es correcto y comprensible.

**Evaluador 3:**

- Rapidez y comodidad
- Facilidad de acceso a la información, sobre todo mejor con GPT-4 que tiene más aciertos y razona mejor las opciones.
- Lenguaje comprensible.

**Evaluador 4:** Más fortalezas con el GPT-4 que parece “más científico” y acierta gran parte de las preguntas, pocos errores. El lenguaje que utiliza es comprensible

**Evaluador 5:**

- Chat-GPT: razona entre lo verdadero y falso en general, lenguaje comprensible
- Chat-GPT4: más aciertos, entendibles, razona lo verdadero y falso en general

**Evaluador 6:**

- Porcentaje muy elevado de aciertos en las preguntas
- Actualizado a nivel bibliográfico
